# Comparison of eight insulin-independent indices for identifying insulin resistance in Chinese young and middle-aged adults without diabetes: favorable performance of METS-IR and TyG-BMI

**DOI:** 10.3389/fmed.2026.1915279

**Published:** 2026-09-07

**Authors:** Jingyang Gao, Yunqiang He, Hemin Jiang, Fangzhou Lu, Juming Tao, Qianhao Xu, Yan Wang, Jiachen Wang, Tao Yang, Qi Fu, Shuai Zheng

**Affiliations:** Department of Endocrinology and Metabolism, The First Affiliated Hospital with Nanjing Medical University, 300 Guangzhou Road, Nanjing, China

**Keywords:** HOMA-IR, insulin resistance, Matsuda index, METS-IR, tyG-BMI

## Abstract

**Background:**

Homeostasis model assessment of insulin resistance (HOMA-IR) mainly reflects basal IR, whereas dynamic measures such as the Matsuda index are less practical.

**Methods:**

This cross-sectional study evaluated eight non-insulin-based metabolic indices [the metabolic score for insulin resistance [METS-IR], triglyceride-glucose body mass index [TyG-BMI], TyG, TyG-WC, LAP, TG/HDL-C, VAI, CMI] as IR surrogates in 710 Chinese adults without diabetes (aged 20–60 years). IR was separately defined using two criteria (1): HOMA-IR above the 75th percentile, and (2) Matsuda index below the 25th percentile, in normal-weight participants with normal glucose tolerance. Correlations, restricted cubic splines, and receiver operating characteristic (ROC) analyses were performed, including separate sex- and 10-year age-stratified subgroup analyses.

**Results:**

METS-IR and TyG-BMI showed moderate correlations with both HOMA-IR and the Matsuda index. METS-IR, TyG, TyG-BMI, and TyG-WC were linearly associated with IR. In ROC analyses, overall AUCs for METS-IR and TyG-BMI were 0.776 and 0.770 for HOMA-IR-defined IR and 0.768 and 0.765 for Matsuda index-defined IR, indicating moderate discrimination. At cut-offs of 35.54 for METS-IR and 211.37 for TyG-BMI (which were consistent across both IR definitions), sensitivities were 66.1–68.9%, and specificities were 75.1–78.4%. Under the HOMA-IR definition, METS-IR had a significantly larger AUC than six indices, but not TyG-BMI. Under the Matsuda definition, its AUC did not differ from those of TyG-BMI, TyG-WC, LAP, or CMI. Rankings varied across subgroups.

**Conclusion:**

METS-IR and TyG-BMI demonstrated comparable and favorable performance, serving as practical, insulin-independent initial screening markers for identifying elevated IR risk in Chinese young and middle-aged adults without diabetes. Rather than replacing insulin measurements, these indices offer valuable risk stratification tools in primary care or epidemiological settings where insulin assays are routinely unavailable.

## 1 Introduction

Insulin resistance (IR) is characterized by reduced responsiveness of target tissues to insulin and compensatory hyperinsulinemia (1, 2). IR is fundamental to the development of type 2 diabetes (T2D) and is common in prediabetes (3). Prolonged IR eventually leads to β-cell decompensation, which is the primary mechanism underlying the progression to T2D (4, 5). IR has also been recognized as a key driver of various metabolic diseases, including obesity, metabolic syndrome (MetS), metabolic dysfunction-associated fatty liver disease (MAFLD), and cardiovascular disease (CVD) (6–9). Early identification of IR and enhancing the availability of IR detection are beneficial for assessing the risk of developing T2D and other metabolic disorders, and are crucial for guiding individualized therapeutic strategies.

The hyperinsulinemic-euglycemic clamp (HEC) is the gold-standard method for assessing IR (10), but its complexity, invasiveness, high cost, and time consumption limit its use mainly to research settings. Consequently, non-invasive surrogate indices such as the homeostasis model assessment of IR (HOMA-IR) and the Matsuda index are commonly employed in clinical practice (11, 12). HOMA-IR mainly reflects hepatic IR, while the Matsuda index, derived from the oral glucose tolerance test (OGTT), assesses both hepatic and peripheral insulin sensitivity (11, 12). Nevertheless, both indices require accurate insulin measurement. These requirements limit scalability in primary care and population screening. Therefore, developing cost-effective and insulin-independent IR indicators is critical to improve clinical screening and early metabolic dysfunction detection.

Several simple and practical indicators have been developed, including metabolic score for IR (METS-IR), triglyceride-glucose (TyG), TyG-body mass index (TyG-BMI), TyG-waist circumference (TyG-WC), lipid accumulation product (LAP), triglyceride to high-density lipoprotein cholesterol (TG/HDL-C) ratio, visceral adiposity index (VAI), and cardiometabolic index (CMI) (13–19). These indices are calculated from routinely available clinical and biochemical parameters.

However, most previous studies have evaluated these indices against HOMA-IR alone, which primarily reflects fasting hepatic IR. Evidence comparing insulin-independent indices against dynamic OGTT-derived insulin sensitivity measures remains limited, particularly in Chinese non-diabetic adults. Focusing on adults without diabetes helps identify IR at an earlier stage before overt diabetes develops. Therefore, this study aimed to compare eight routinely available insulin-independent metabolic indices against both HOMA-IR and the Matsuda index, and to explore which indices might serve as practical initial screening tools for IR in non-diabetic Chinese adults aged 20–60 years.

## 2 Methods

### 2.1 Study design and participants

A total of 853 individuals aged 20–70 years residing in Jurong City, China, were initially recruited from the same cohort reported in our prior publication (20). All subjects were ethnic Han Chinese. After excluding 27 participants aged 61–70 years, 77 participants with previously or newly diagnosed diabetes, and 39 participants with incomplete insulin measurements across the five OGTT time points, a final sample of 710 participants was included in the analysis. Criteria for exclusion of diabetes included a previous diagnosis of diabetes according to the American Diabetes Association criteria (21). The cohort was grouped by age with 10-year intervals: 20–29 years (*n* = 167), 30–39 years (*n* = 213), 40–49 years (*n* = 143), and 50–60 years (*n* = 187). All participants provided written informed consent before inclusion in the study. The study protocol was approved by the Institutional Review Board of the First Affiliated Hospital with Nanjing Medical University (2021-SR-298).

### 2.2 Data collection

Demographic characteristics were obtained using a comprehensive health and lifestyle questionnaire, including age, sex, current smoker and current drinker status, medical history, and current medication use. Participants were categorized as current smokers or non-current smokers. Current drinkers were defined as those who consumed alcohol at least once per week during the preceding six months.

Anthropometric assessments and blood sampling were performed following an overnight fast of at least 12 h. Standardized methods were used to measure height, weight, and waist circumference (WC). Body mass index (BMI) was calculated as weight in kilograms divided by the square of height in meters (kg/m^2^). Blood pressure (BP) was measured twice to obtain average readings after a minimum of 5 min of rest. Participants underwent a standard 75-g OGTT, with blood samples obtained at 0, 30, 60, 120, and 180 min to assess glucose and insulin.

Insulin levels were measured using chemiluminescence detection kits (YHLO Biotech, Shenzhen, China). Fasting plasma glucose (FPG) was analyzed by the hexokinase method (AU5400; Olympus, Tokyo, Japan). Glycated hemoglobin (HbA1c) was assessed using capillary blood samples (Variant II; Bio-Rad, Hercules, USA). Serum levels of triglycerides (TG), low-density lipoprotein cholesterol (LDL-C), high-density lipoprotein cholesterol (HDL-C), and total cholesterol (TC) were quantified using a chemiluminescence autoanalyzer (Modular E170; Roche Diagnostics, Basel, Switzerland).

### 2.3 Definition of IR

There are no universally accepted optimal cut-off values for HOMA-IR and the Matsuda index for diagnosing IR in Chinese adults. Furthermore, absolute insulin values can vary significantly depending on the laboratory assays used. To minimize potential misclassification bias, we derived cohort-specific thresholds from a metabolically low-risk internal reference group comprising participants with normal glucose tolerance and a BMI < 24 kg/m^2^. IR was then separately defined using two criteria: (1) HOMA-IR ≥ 3.17, corresponding to the 75th percentile, and (2) Matsuda index ≤ 3.33, corresponding to the 25th percentile, in this reference population (22). These thresholds were applied uniformly in the overall, sex-stratified, and age-stratified analyses. To assess the robustness of the findings to the cohort-specific definition, a sensitivity analysis was performed using a published HOMA-IR threshold of 2.69 (23).

HOMA-IR = [FPG (mmol/L) × fasting insulin (*μ*IU/mL)]/22.5 (12)

Matsuda index = 10,000/ {[FPG (mg/dL) × fasting insulin (μIU/mL)] × [mean OGTT glucose (mg/dL) × mean OGTT insulin (μIU/mL)]}^0.5^ (11)

The Matsuda index can be calculated using three-point (0, 60, and 120 min), four-point (0, 30, 60, and 120 min), or all five-point (0, 30, 60, 120, and 180 min) OGTT, and the latter two indices have been shown to correlate strongly with HOMA-IR (Supplementary Figure 1). Considering the practicality in real-world applications, the present study used the four-point method for calculating the Matsuda index.

### 2.4 Calculation of IR surrogate indices

Eight widely used IR surrogate indices were calculated using the following formulas:

METS-IR = {Ln [2 × FPG (mg/dL) + TG (mg/dL)] × BMI (kg/m^2^)}/Ln [HDL-C (mg/dL)] (13)

TyG = Ln [TG (mg/dL) × FPG (mg/dL)/2] (14)

TyG-BMI = Ln [TG (mg/dL) × FPG (mg/dL)/2] × BMI (kg/m^2^) (15)

TyG-WC = Ln [TG (mg/dL) × FPG (mg/dL)/2] × WC (cm) (15)

LAP for men = [WC (cm)−65] × TG (mmol/L) (17)

LAP for women = [WC (cm)−58] × TG (mmol/L) (17)

TG/HDL-C = TG (mmol/L)/HDL-C (mmol/L) (16)

VAI for men = {WC (cm)/[39.68 + 1.88 × BMI (kg/m^2^)]} × [TG (mmol/L)/1.03] × [1.31/HDL-C (mmol/L)] (18)

VAI for women = {WC (cm)/[36.58 + 1.89 × BMI (kg/m^2^)]} × [TG (mmol/L)/0.81] × [1.52/HDL-C (mmol/L)] (18)

CMI = [TG (mmol/L)/HDL-C (mmol/L)] × [WC (cm)/height (cm)] (19)

### 2.5 Statistical analysis

Statistical analyses were performed using SPSS version 26.0 (IBM Corp., Armonk, NY, USA) and R Version 4.5.1 (R Foundation for Statistical Computing, Vienna, Austria). Normality was assessed using the Shapiro–Wilk test. Because all continuous variables exhibited non-normal distributions, they were presented as medians with interquartile ranges (IQRs) and were compared using the Mann–Whitney U test. Categorical variables were expressed as counts (percentages) and were analyzed using the chi-square test. The chi-square test for trend was applied to evaluate the linear trend of IR prevalence across ordered age groups. Spearman's rank correlation evaluated associations between each surrogate index and HOMA-IR and the Matsuda index. Restricted cubic spline (RCS) regression models with 4 knots were applied to evaluate potential dose–response relationships between each surrogate index and the odds of IR. These models were adjusted for age, sex, current smoker and current drinker status, SBP, DBP, and antihypertensive and lipid-lowering medication use. Receiver operating characteristic (ROC) curve analysis evaluated the discriminative ability of surrogate indices for IR, with differences in area under the curve (AUC) compared using DeLong's test. Cut-offs were determined by maximizing the Youden index. A two-sided *P* < 0.05 was considered statistically significant.

## 3 Results

### 3.1 Baseline characteristics

Among the 710 participants included in the final analysis, 416 (58.6%) had normal glucose tolerance, and 294 (41.4%) had prediabetes. When IR was defined as HOMA-IR ≥ 3.17, the prevalence of IR was 49.7% (*n* = 197) in men and 38.9% (*n* = 122) in women. Similarly, utilizing a Matsuda index cut-off of 3.33 to define IR, the prevalence of IR among men and women was 54.5% (*n* = 216) and 38.5% (*n* = 121), respectively. IR prevalence decreased across ordered 10-year age groups for both definitions (HOMA-IR: 50.9%, 47.4%, 46.9%, and 35.3%; chi-square test for trend: *P* = 0.003; Matsuda index: 53.3%, 48.8%, 48.3%, and 40.1%; *P* = 0.015; Supplementary Table 1).

Table 1 shows the general demographic and clinical characteristics of the study population, stratified by gender. Overall, men had significantly higher values than women in most anthropometric and metabolic parameters, including BMI, WC, SBP, DBP, FPG, 2h-PG, HbA1c, TG, and LDL-C (all *P* < 0.05). Similarly, IR-related indices, such as HOMA-IR, METS-IR, TyG, TyG-BMI, TyG-WC, LAP, TG/HDL-C ratio, VAI, and CMI, were all significantly higher in men than in women. In contrast, women had higher levels of the Matsuda index and HDL-C (both *P* < 0.001). Moreover, the proportions of current smokers and drinkers were markedly higher in men than in women (both *P* < 0.001). The use of antihypertensive medications was also more frequent in men (*P* = 0.007), while there was no significant sex difference in the use of lipid-lowering agents.

**Table 1:** Characteristics of the participants.

Characteristic	Total	Men	Women	*P* value
	(*n* = 710)	(*n* = 396)	(*n* = 314)	
Age (years)	39.0 (30.0–50.0)	40.0 (31.0–52.0)	36.0 (29.0–48.0)	0.001
BMI, kg/m^2^	24.08 (21.75–26.61)	25.04 (22.84–27.42)	22.62 (20.79–25.04)	< 0.001
WC, m	0.82 (0.75–0.91)	0.88 (0.81–0.94)	0.76 (0.71–0.82)	< 0.001
SBP, mmHg	121.0 (112.0–132.0)	127.0 (119.0–136.5)	113.5 (106.5–122.0)	< 0.001
DBP, mmHg	82.0 (75.5–89.0)	85.8 (79.0–92.5)	77.5 (72.0–83.5)	< 0.001
FPG, mmol/L	5.64 (5.35–6.02)	5.82 (5.48–6.18)	5.51 (5.21–5.76)	< 0.001
2h-PG, mmol/L	6.24 (5.37–7.33)	6.41 (5.39–7.54)	6.12 (5.36–7.10)	0.012
HbA1c, %	5.40 (5.20–5.60)	5.40 (5.20–5.70)	5.30 (5.10–5.50)	< 0.001
TG, mmol/L	1.12 (0.74–1.63)	1.30 (0.87–1.99)	0.88 (0.67–1.30)	< 0.001
LDL-C, mmol/L	2.98 (2.49–3.53)	3.14 (2.64–3.70)	2.81 (2.40–3.30)	< 0.001
HDL-C, mmol/L	1.48 (1.25–1.71)	1.35 (1.16–1.58)	1.61 (1.44–1.82)	< 0.001
HOMA-IR	2.99 (2.23–4.13)	3.17 (2.31–4.53)	2.79 (2.15–3.66)	0.003
Matsuda index	3.40 (2.54–4.64)	3.20 (2.29–4.47)	3.68 (2.83–4.79)	< 0.001
METS-IR	34.32 (29.76–39.64)	36.98 (31.96–43.03)	30.85 (27.53–35.38)	< 0.001
TyG	8.54 (8.09–8.94)	8.72 (8.32–9.14)	8.26 (7.94–8.67)	< 0.001
TyG-BMI	204.88 (179.22–237.95)	221.84 (191.90–252.77)	188.22 (168.25–214.24)	< 0.001
TyG-WC	699.91 (618.95–807.82)	768.59 (687.98–858.59)	625.90 (574.85–697.37)	< 0.001
LAP	21.91 (11.85–44.63)	29.27 (15.57–55.34)	15.38 (9.10–27.47)	< 0.001
TG/HDL-C	1.71 (1.04–2.83)	2.18 (1.35–3.81)	1.23 (0.89–1.94)	< 0.001
VAI	1.10 (0.70–1.83)	1.20 (0.74–2.17)	0.95 (0.67–1.49)	< 0.001
CMI	0.37 (0.21–0.66)	0.49 (0.29–0.93)	0.25 (0.18–0.42)	< 0.001
Current smokers [n (%)]	217 (30.6)	215 (54.3)	2 (0.6)	< 0.001
Current drinkers [n (%)]	206 (29.0)	185 (46.7)	21 (6.7)	< 0.001
Lipid-lowering medication use [n (%)]	7 (1.0)	5 (1.3)	2 (0.6)	0.473
Antihypertensive medication use [n (%)]	50 (7.0)	37 (9.3)	13 (4.1)	0.007

Data are presented as median (IQR) or number (%). *P* values were calculated using the Mann–Whitney U test for continuous variables and the chi-square test for categorical variables. BMI, body mass index; WC, waist circumference; SBP, systolic blood pressure; DBP, diastolic blood pressure; FPG, fasting plasma glucose; 2h-PG, 2-hour post-load plasma glucose; HbA1c, glycated hemoglobin; TG, triglycerides; LDL-C, low-density lipoprotein cholesterol; HDL-C, high-density lipoprotein cholesterol; HOMA-IR, homeostasis model assessment of insulin resistance; METS-IR, metabolic score for insulin resistance; TyG, triglyceride–glucose; TyG-BMI, TyG-body mass index; TyG-WC, TyG-waist circumference; LAP, lipid accumulation product; TG/HDL-C, triglyceride-to-HDL-C ratio; VAI, visceral adiposity index; CMI, cardiometabolic index.

### 3.2 Spearman's rank correlations of IR surrogate indices with HOMA-IR and the Matsuda index

**Figure 1:**
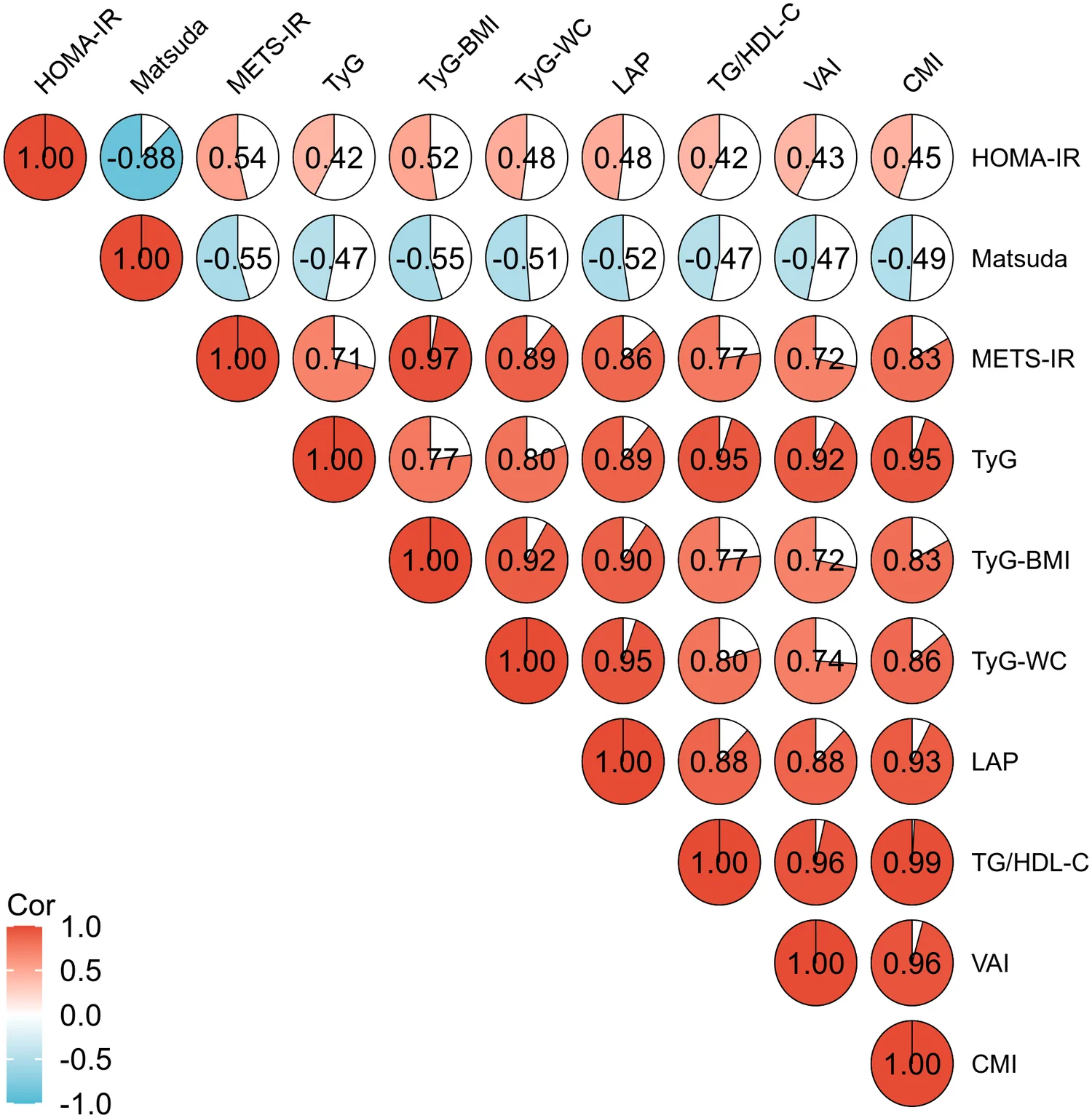
Spearman's rank correlations of IR surrogate indices with HOMA-IR and the matsuda index. HOMA-IR, homeostasis model assessment of insulin resistance; METS-IR, metabolic score for insulin resistance; TyG, triglyceride–glucose; TyG-BMI, TyG-body mass index; TyG-WC, TyG-waist circumference; LAP, lipid accumulation product; TG/HDL, triglyceride-to–HDL-C ratio; VAI, visceral adiposity index; CMI, cardiometabolic index.

Figure 1 presents Spearman's rank correlation between eight surrogate indices of IR and two reference indices, HOMA-IR and the Matsuda index. As expected, all surrogate indices showed positive correlations with HOMA-IR and inverse correlations with the Matsuda index. Among them, METS-IR (*ρ* = 0.54 with HOMA-IR, *ρ* = −0.55 with the Matsuda index) and TyG-BMI (*ρ* = 0.52 with HOMA-IR, *ρ* = −0.55 with the Matsuda index) exhibited moderate correlations with both reference measures. Subgroup analyses were performed separately by sex and by 10-year age group. METS-IR and TyG-BMI generally maintained moderate correlations across most subgroups. Additionally, when the Matsuda index was used as the reference, comparable correlation coefficients were also observed for LAP in the 20–29 and 30–39 years groups, and for TyG-WC in the 30–39 and 40–49 years groups, as well as in women (Supplementary Table 2).

### 3.3 Dose–response relationships between IR surrogate indices and IR defined using HOMA-IR or the Matsuda index

**Figure 2:**
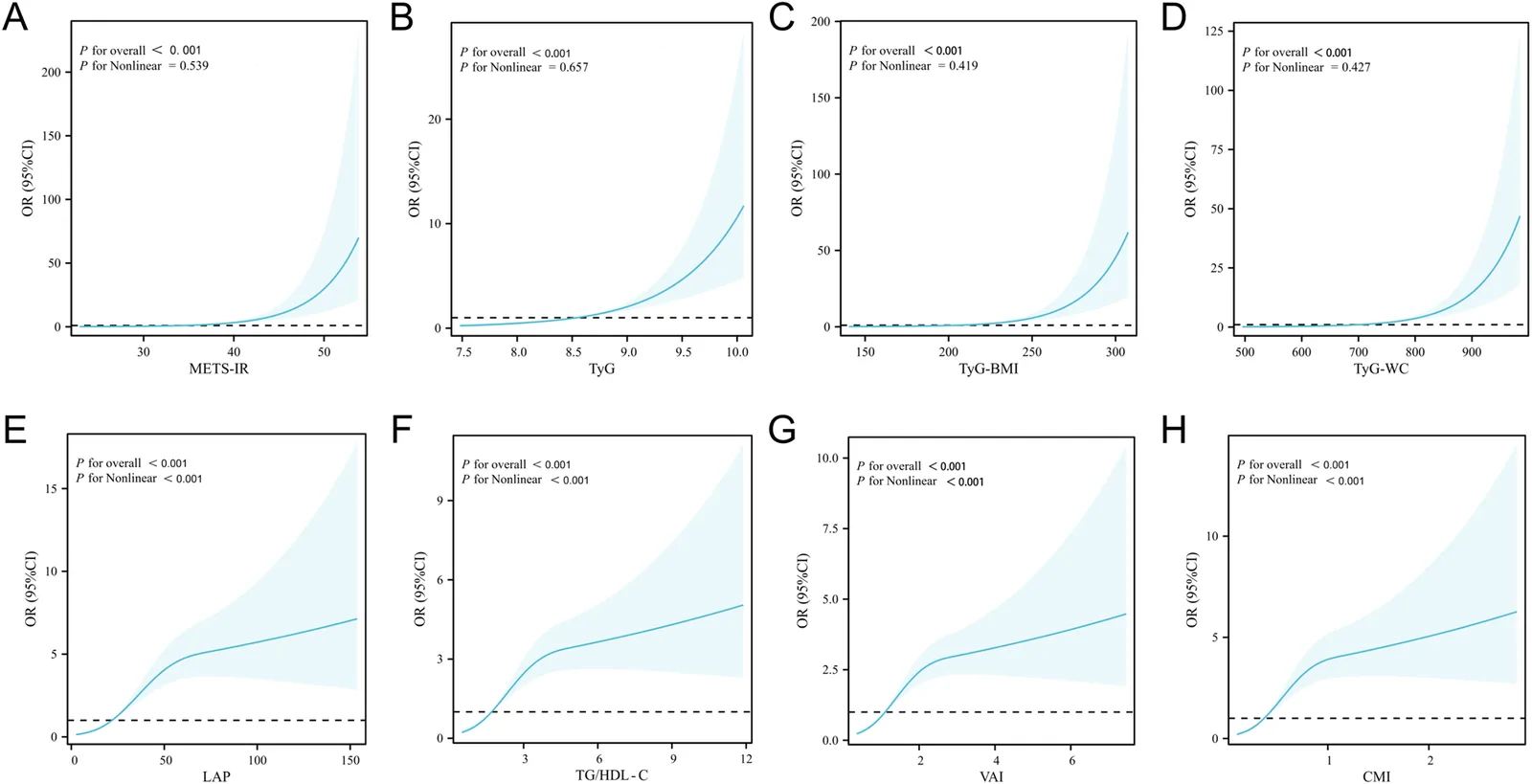
Dose–response relationships between IR surrogate indices and IR defined using HOMA-IR. Restricted cubic splines were used to evaluate dose–response relationships between IR indicated by HOMA-IR and the following: METS-IR **(A)**, TyG **(B)**, TyG-BMI **(C)**, TyG-WC **(D)**, LAP **(E)**, TG/HDL-C **(F)**, VAI **(G)**, and CMI **(H)** The model was adjusted for age, sex, current smoker and drinker status, SBP, DBP, and antihypertensive and lipid-lowering medication use.

As shown in Figure 2, RCS analyses were conducted to evaluate the dose–response relationships between each surrogate index and the odds of IR defined by HOMA-IR. All indices demonstrated significant associations (*P* < 0.001 overall). Specifically, METS-IR, TyG, TyG-BMI, and TyG-WC showed predominantly linear trends (*P* for nonlinea*r* = 0.539, 0.657, 0.419, and 0.427, respectively), with a continuous increase in odds ratios of IR. The RCS models using the Matsuda index definition showed similar trends for METS-IR, TyG, TyG-BMI, and TyG-WC (Supplementary Figure 2). In contrast, LAP, TG/HDL-C, VAI, and CMI displayed significant associations, but with nonlinear trends.

### 3.4 ROC evaluation identifies favorable overall discrimination by METS-IR and TyG-BMI

**Figure 3:**
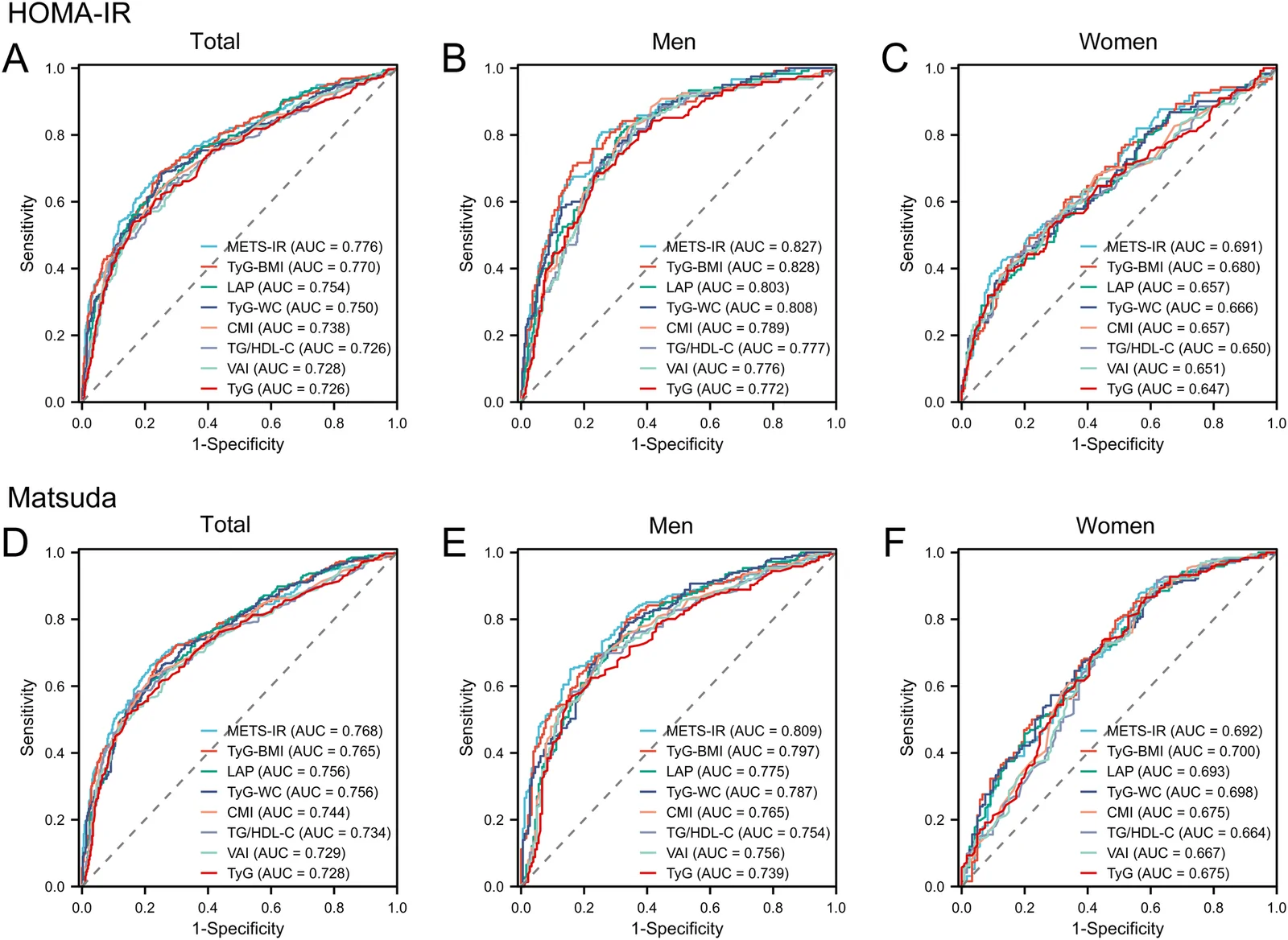
Comparison of the discriminative performance of IR surrogate indices. Comparison of the discriminative performance of eight surrogate indices for IR using ROC analysis, with HOMA-IR as the reference standard **(A–C)** and the Matsuda index as the reference standard **(D–F)**, in total and sex-specific groups.

ROC analysis was performed for the total population and sex-specific subgroups to evaluate the discriminative performance of eight IR surrogate indices against HOMA-IR-defined IR (Figures 3A–C). In the total population, METS-IR and TyG-BMI yielded the two highest AUCs (0.776, 95% confidence interval [CI]: 0.741–0.810; and 0.770, 95% CI: 0.734–0.805, respectively; Table 2). Their AUCs did not differ significantly (*P* = 0.247), whereas METS-IR had a significantly higher AUC than each of the remaining six indices (all *P* ≤ 0.049). The optimal cut-off value for METS-IR was 35.54 (sensitivity 67.3%, specificity 77.4%), while TyG-BMI had a cut-off of 211.37 (sensitivity 68.9%, specificity 75.1%). Similar patterns were observed in sex-stratified analyses. In men, both METS-IR and TyG-BMI achieved AUCs above 0.80 (0.827 and 0.828, respectively). In women, METS-IR (AUC = 0.691) and TyG-BMI (AUC = 0.680) remained the top-performing indices. Across age groups, METS-IR and TyG-BMI generally remained among the leading indices, with the highest AUCs observed in the 40–49-year group; TyG-WC and LAP were also competitive, while in the 50–60-year group, METS-IR significantly outperformed TyG, TG/HDL-C, VAI, and CMI, but not TyG-BMI, TyG-WC, or LAP (Supplementary Table 3).

**Table 2:** Comparison of eight IR surrogate indices for identifying IR defined separately by HOMA-IR and the matsuda index in the overall population.

Index	AUC (95% CI)	*P* value vs. METS-IR	Cut-off	Sensitivity	Specificity
HOMA-IR-defined IR					
METS-IR	0.776 (0.741–0.810)	-	35.54	0.673	0.774
TyG-BMI	0.770 (0.734–0.805)	0.247	211.37	0.689	0.751
TyG-WC	0.750 (0.714–0.787)	0.010	720.61	0.685	0.748
LAP	0.754 (0.718–0.790)	0.049	30.16	0.590	0.815
TyG	0.726 (0.686–0.763)	0.001	8.79	0.539	0.839
TG/HDL-C	0.726 (0.687–0.763)	< 0.001	1.88	0.649	0.729
VAI	0.728 (0.691–0.766)	0.003	1.60	0.505	0.861
CMI	0.738 (0.700–0.775)	0.002	0.44	0.637	0.756
Matsuda index-defined IR					
METS-IR	0.768 (0.733–0.803)	-	35.54	0.661	0.784
TyG-BMI	0.765 (0.730–0.800)	0.465	211.37	0.673	0.757
TyG-WC	0.756 (0.721–0.792)	0.238	719.84	0.663	0.747
LAP	0.756 (0.721–0.792)	0.295	28.42	0.606	0.795
TyG	0.728 (0.690–0.765)	0.012	8.81	0.516	0.858
TG/HDL-C	0.734 (0.696–0.771)	0.017	2.21	0.576	0.834
VAI	0.729 (0.692–0.766)	0.015	1.19	0.645	0.728
CMI	0.744 (0.707–0.780)	0.054	0.44	0.627	0.784

IR was separately defined using two criteria: (1) HOMA-IR ≥ 3.17, corresponding to the 75th percentile, and (2) Matsuda index ≤ 3.33, corresponding to the 25th percentile, in the overall reference group of normal-weight participants with normal glucose tolerance. *P* values are from DeLong's nonparametric test comparing each AUC with that of METS-IR. Cut-offs were determined by maximizing the Youden index. AUC, area under the receiver operating characteristic curve; CI, confidence interval; METS-IR, metabolic score for insulin resistance; TyG, triglyceride-glucose; TyG-BMI, TyG-body mass index; TyG-WC, TyG-waist circumference; LAP, lipid accumulation product; TG/HDL-C, triglyceride to high-density lipoprotein cholesterol; VAI, visceral adiposity index; CMI, cardiometabolic index.

In the sensitivity analysis using HOMA-IR ≥ 2.69, METS-IR and TyG-BMI remained the two highest-ranking indices in the total population, with AUCs of 0.748 and 0.738, respectively, indicating a broadly consistent ranking (Supplementary Figure 3).

ROC analysis using the Matsuda index-defined IR showed generally consistent results. In the total population, METS-IR exhibited the highest AUC of 0.768 (95% CI: 0.733–0.803), closely followed by TyG-BMI (AUC: 0.765, 95% CI: 0.730–0.800). However, DeLong's test indicated no statistically significant differences in AUCs among METS-IR, TyG-BMI, TyG-WC, LAP and CMI (Table 2). In sex-stratified analyses, METS-IR and TyG-BMI continued to serve as the leading indicators in men; in women, TyG-BMI and TyG-WC achieved the highest AUCs, with METS-IR and LAP showing comparable discriminative values (Figures 3D–F). In age-stratified analyses, LAP and TyG-WC showed competitive performance in some younger groups, whereas most significant differences were observed in the 50–60-year group, where METS-IR outperformed all indices except TyG-WC; TyG-BMI outperformed METS-IR in the 20–29-year group (Supplementary Table 4).

## 4 Discussion

The present study systematically evaluated eight insulin-independent surrogate indices for identifying IR in Chinese non-diabetic adults aged 20–60 years. Among all evaluated indices, METS-IR and TyG-BMI consistently showed moderate correlations with both HOMA-IR and the OGTT-derived Matsuda index. In addition, both indices showed approximately linear dose–response relationships with IR risk in RCS analyses and achieved favorable overall discriminative performance in ROC analyses. Taken together, METS-IR and TyG-BMI showed comparatively favorable and consistent performance across both IR definitions and most subgroups, although TyG-WC and LAP were competitive in selected subgroups, particularly under the Matsuda index definition.

The relative advantage of METS-IR and TyG-BMI was more evident when HOMA-IR was used as the reference standard, whereas no significant AUC differences were observed among METS-IR, TyG-BMI, TyG-WC, CMI and LAP under the Matsuda index definition. This may be because HOMA-IR mainly reflects fasting hepatic IR, while the Matsuda index incorporates dynamic OGTT-derived glucose-insulin responses and provides a broader assessment of whole-body insulin sensitivity. Accordingly, their advantage over other adiposity-related indices should be interpreted cautiously when IR is defined by the Matsuda index.

An additional methodological strength was the availability of full OGTT data. This enabled evaluation of the eight surrogate indices against the four-point Matsuda index, an OGTT-derived measure of hepatic and peripheral insulin sensitivity, in addition to HOMA-IR. However, evidence evaluating routinely available insulin-independent indices against the Matsuda index in Chinese adults without diabetes remains limited. Our study extends previous work by comparing eight such indices against both HOMA-IR and the four-point Matsuda index in a non-diabetic Chinese cohort.

Previous studies have reported favorable associations or predictive performance of METS-IR and TyG-BMI for diabetes, atherosclerosis, MAFLD, and other metabolic disease outcomes (24–29). A recent prospective cohort study conducted in Chinese and Japanese populations similarly demonstrated that METS-IR and TyG-BMI were effective surrogate markers for predicting incident diabetes, further supporting the applicability of these indices in East Asian populations (30). These findings suggest that indices integrating multiple metabolic abnormalities may better reflect the complex pathophysiology of IR.

The favorable performance of METS-IR may reflect its integration of FPG, TG, HDL-C, and BMI, thereby capturing complementary aspects of glucose-lipid metabolism and adiposity. Its reported associations with fasting insulin and visceral, intrahepatic, and pancreatic fat further support this interpretation (13). Prospective studies have linked METS-IR to incident T2D (13), reported comparable or better performance than TyG-derived and other non-insulin indices (31), and demonstrated correlations with clamp-derived insulin sensitivity (32), supporting its potential utility when insulin assays are unavailable.

TyG-BMI incorporates BMI into TyG and has been proposed as a practical surrogate for IR (15). In our study, it showed similar overall discrimination to METS-IR and outperformed TyG alone, suggesting that the inclusion of adiposity information may improve the ability of TyG-based indices to capture systemic IR. However, TyG-WC showed comparable performance in selected sex- and age-specific analyses under the Matsuda index definition, indicating that BMI and waist circumference may reflect different dimensions of adiposity-related metabolic dysfunction. The integration of glucose-lipid metabolism with overall adiposity may partly explain the favorable performance of TyG-BMI (15, 33), consistent with previous reports linking it to IR, diabetes, and cardiovascular disease (30, 34).

The ROC-derived METS-IR cut-off in our population was 35.54, which differed from previously reported cut-offs of 31.84 for identifying IR in children and adolescents and 39.77 for identifying MAFLD in NHANES participants (35, 36). Given substantial variations in population profiles and outcome definitions, population- and outcome-specific cut-offs may be more appropriate than applying a universal cut-off. Notably, the ROC-derived cut-offs for METS-IR and TyG-BMI in the overall population were identical when IR was defined by HOMA-IR and the Matsuda index. However, because the primary HOMA-IR and Matsuda index thresholds were derived from an internal low-risk reference subgroup, internal optimism and limited generalizability cannot be excluded. Sensitivity analysis using a published HOMA-IR threshold of 2.69 yielded a broadly similar ranking, whereas no alternative Matsuda threshold was examined because no universally accepted threshold exists. For example, a recent study of 93 non-diabetic young adult men used the OGTT-derived Matsuda index as the reference measure and identified a cut-off of 4.03 for defining IR (37), highlighting potential population-specific variation in Matsuda index thresholds. Accordingly, the HOMA-IR and Matsuda index thresholds used in our study should be regarded as operational definitions of IR, whereas the ROC-derived cut-offs for the insulin-independent indices should be interpreted as candidate screening cut-offs rather than diagnostic thresholds.

In our study, IR prevalence was higher and insulin sensitivity was lower in men than in women, indicating sex-related differences in IR. This finding is consistent with previous evidence that sex hormones influence adipose tissue distribution and systemic metabolic regulation (38). Sex-stratified analyses further showed that METS-IR and TyG-BMI had stronger discriminative performance in men than in women. This difference may partly reflect sex-related variations in adipose tissue distribution and ectopic lipid accumulation (39), although the lack of direct body-composition measurements limits mechanistic interpretation. Women tend to preferentially store fat in subcutaneous depots, partly owing to estrogen-related effects on adipose tissue distribution and insulin sensitivity (38), which may reduce the sensitivity of these indices to IR-related metabolic abnormalities. Age-stratified analyses showed broadly favorable performance of METS-IR and TyG-BMI across age groups, although rankings varied and the highest AUCs were observed in participants aged 40–49 years. These exploratory subgroup findings require confirmation in larger independent cohorts.

In addition to METS-IR and TyG-BMI, the other IR surrogate indices, including LAP, TG/HDL-C, VAI, and CMI, also demonstrated significant associations with IR risk. LAP demonstrated relatively favorable performance in selected analyses, particularly in some age- and sex-specific subgroups when the Matsuda index was used as the reference standard, which is consistent with previous evidence suggesting that the combination of waist circumference and triglycerides may effectively reflect visceral lipid accumulation and metabolic dysfunction (17). Similarly, TG/HDL-C, VAI, and CMI are established markers of dyslipidemia and visceral adiposity and have been associated with IR and cardiometabolic risk in previous studies (18, 37, 40). Nevertheless, their discriminative performance in the present study was generally lower than that of METS-IR and TyG-BMI, possibly because these indices mainly emphasize lipid abnormalities or body fat distribution, whereas IR reflects complex interacting disturbances in glucose metabolism, adiposity, ectopic fat accumulation, and systemic metabolic dysfunction (41). Therefore, while they may not serve as preferred primary screening tools for early IR, these indices remain valuable complementary instruments for evaluating specific visceral fat dysfunctions and downstream cardiovascular risks in targeted clinical scenarios.

This study has several notable strengths. First, the study focused on adults without diabetes, a population in whom practical identification of IR may be particularly relevant for early prevention of diabetes and cardiometabolic disease. Second, the availability of dynamic OGTT-derived data enabled evaluation against the Matsuda index in addition to HOMA-IR. Third, the 10-year age-stratified analyses showed that the favorable performance of METS-IR and TyG-BMI was generally consistent across age groups. Finally, both indices can be calculated from routine clinical parameters and do not require insulin assays, making them suitable for primary care and health check-up settings.

Several limitations should be acknowledged. First, the modest sample size and single-center recruitment of Han Chinese participants from Jurong City may limit generalizability. Second, the primary HOMA-IR and Matsuda index thresholds were derived from an internal low-risk reference subgroup, introducing possible internal optimism. Sensitivity analysis using HOMA-IR ≥ 2.69 produced a broadly similar ranking, but no alternative Matsuda index threshold was examined. Third, the lack of HEC measurements limits the ability to directly validate these surrogate indices. Even in a subset of participants, HEC measurements could have enabled direct validation against the gold standard. Fourth, direct measures of body composition, visceral adiposity, hepatic fat, and physical activity were unavailable; therefore, unmeasured differences in body fat distribution and lifestyle may have contributed to residual confounding, particularly for indices incorporating BMI or waist circumference. Finally, due to the cross-sectional design, we were unable to determine whether METS-IR or TyG-BMI could predict incident diabetes or future cardiometabolic outcomes.

## 5 Conclusion

In conclusion, METS-IR and TyG-BMI showed comparatively favorable and consistent performance among eight insulin-independent surrogate indices for identifying IR in Chinese non-diabetic adults aged 20–60 years. These simple, non-invasive, and cost-effective indicators can be readily calculated from routinely available clinical parameters. Their consistent performance across HOMA-IR and the OGTT-derived Matsuda index supports their potential utility as practical initial screening tools for IR in epidemiological studies, health check-up programs, and primary care settings where insulin measurements are not routinely available. Although they cannot substitute for direct insulin testing or comprehensive clinical evaluations, further prospective studies with external validation and clamp-based assessment are warranted to confirm their clinical applicability.

## Data Availability

The datasets generated during and/or analyzed during the current study are not publicly available but are available from the corresponding author upon reasonable request. Requests to access the datasets should be directed to Shuai Zheng, zhengshuai89@njmu.edu.cn.
