# Supplementary material for: Comparison of eight insulin-independent indices for identifying insulin resistance in Chinese young and middle-aged adults without diabetes: favorable performance of METS-IR and TyG-BMI
Source: Front Med (Lausanne). 2026 Sep 7;13:1915279. doi: 10.3389/fmed.2026.1915279 (PMC13590358; doi:10.3389/fmed.2026.1915279)
Supplement: Supplementary file 1 [file Datasheet1.docx]

Supplementary Material

# 1 Supplementary Tables

## 1.1 Supplementary Table 1. Prevalence of IR defined separately by HOMA-IR and the Matsuda index across sex and 10-year age groups.

| Variable | Total  (n=710) | Men (n=396) | Women (n=314) | 20–29 years (n=167) | 30–39 years (n=213) | 40–49 years (n=143) | 50–60 years (n=187) | *P* for trend |
| --- | --- | --- | --- | --- | --- | --- | --- | --- |
| IR defined by HOMA-IR | 319  (44.9%) | 197  (49.7%) | 122  (38.9%) | 85  (50.9%) | 101  (47.4%) | 67  (46.9%) | 66  (35.3%) | 0.003 |
| IR defined by the Matsuda index | 337  (47.5%) | 216  (54.5%) | 121  (38.5%) | 89  (53.3%) | 104  (48.8%) | 69  (48.3%) | 75  (40.1%) | 0.015 |

IR was separately defined using two criteria: (1) HOMA-IR ≥ 3.17, corresponding to the 75th percentile, and (2) Matsuda index ≤ 3.33, corresponding to the 25th percentile, in the overall reference group of normal-weight participants with normal glucose tolerance. *P* for trend across the four ordered age groups was calculated using the chi-square test for trend.

## 1.2 Supplementary Table 2. Spearman rank correlations of IR surrogate indices with HOMA-IR and the Matsuda index in sex- and age-stratified subgroups.

|  | METS-IR | TyG | TyG-BMI | TyG-WC | LAP | TG/HDL-C | VAI | CMI |
| --- | --- | --- | --- | --- | --- | --- | --- | --- |
| HOMA-IR |  |  |  |  |  |  |  |  |
| 20–29 years  (n=167) | 0.574 | 0.450 | 0.589 | 0.503 | 0.536 | 0.417 | 0.451 | 0.468 |
| 30–39 years  (n=213) | 0.611 | 0.491 | 0.593 | 0.569 | 0.559 | 0.492 | 0.491 | 0.525 |
| 40–49 years  (n=143) | 0.602 | 0.542 | 0.623 | 0.610 | 0.607 | 0.536 | 0.557 | 0.563 |
| 50–60 years  (n=187) | 0.543 | 0.346 | 0.515 | 0.452 | 0.429 | 0.330 | 0.313 | 0.365 |
| Men (n=396) | 0.633 | 0.469 | 0.619 | 0.575 | 0.542 | 0.478 | 0.482 | 0.508 |
| Women (n=314) | 0.400 | 0.308 | 0.390 | 0.362 | 0.333 | 0.287 | 0.287 | 0.307 |
| Matsuda index | | | | | | | | |
| 20–29 years  (n=167) | −0.538 | −0.491 | −0.578 | −0.493 | −0.557 | −0.432 | −0.486 | −0.475 |
| 30–39 years  (n=213) | −0.613 | −0.555 | −0.610 | −0.626 | −0.618 | −0.555 | −0.545 | −0.589 |
| 40–49 years  (n=143) | −0.649 | −0.590 | −0.674 | −0.676 | −0.672 | −0.594 | −0.605 | −0.621 |
| 50–60 years  (n=187) | −0.559 | −0.385 | −0.543 | −0.472 | −0.468 | −0.371 | −0.354 | −0.400 |
| Men (n= 396) | −0.654 | −0.512 | −0.650 | −0.617 | −0.594 | −0.524 | −0.528 | −0.553 |
| Women (n=314) | −0.374 | −0.353 | −0.384 | −0.377 | −0.364 | −0.323 | −0.322 | −0.335 |

Values are Spearman rank correlation coefficients (ρ); all *P* values were < 0.001. HOMA-IR, homeostasis model assessment of insulin resistance; METS-IR, metabolic score for insulin resistance; TyG, triglyceride–glucose; TyG-BMI, TyG-body mass index; TyG-WC, TyG-waist circumference; LAP, lipid accumulation product; TG/HDL-C, triglyceride-to-HDL-C ratio; VAI, visceral adiposity index; CMI, cardiometabolic index.

## 1.3 Supplementary Table 3. Comparison of eight IR surrogate indices for identifying HOMA-IR-defined IR across age groups.

| Index | AUC (95% CI) | *P* value vs METS-IR | Cut-off | Sensitivity | Specificity |
| --- | --- | --- | --- | --- | --- |
| 20–29 years (n=167) | | | | | |
| METS-IR | 0.783 (0.713–0.854) | - | 32.00 | 0.706 | 0.768 |
| TyG-BMI | 0.791 (0.722–0.860) | 0.488 | 193.21 | 0.682 | 0.841 |
| TyG-WC | 0.752 (0.677–0.827) | 0.164 | 692.03 | 0.565 | 0.866 |
| LAP | 0.779 (0.708–0.850) | 0.869 | 11.21 | 0.812 | 0.659 |
| TyG | 0.725 (0.647–0.802) | 0.112 | 8.36 | 0.635 | 0.756 |
| TG/HDL-C | 0.705 (0.625–0.785) | 0.015 | 1.48 | 0.635 | 0.756 |
| VAI | 0.733 (0.657–0.810) | 0.152 | 0.96 | 0.659 | 0.780 |
| CMI | 0.733 (0.656–0.810) | 0.077 | 0.23 | 0.753 | 0.671 |
| 30–39 years (n=213) | | | | | |
| METS-IR | 0.804 (0.745–0.862) | - | 33.44 | 0.762 | 0.712 |
| TyG-BMI | 0.798 (0.740–0.859) | 0.466 | 204.88 | 0.713 | 0.766 |
| TyG-WC | 0.784 (0.722–0.847) | 0.210 | 709.67 | 0.690 | 0.820 |
| LAP | 0.782 (0.720–0.843) | 0.240 | 18.70 | 0.750 | 0.721 |
| TyG | 0.746 (0.681–0.816) | 0.050 | 8.64 | 0.624 | 0.821 |
| TG/HDL-C | 0.751 (0.685–0.817) | 0.036 | 1.88 | 0.653 | 0.768 |
| VAI | 0.755 (0.690–0.821) | 0.083 | 1.08 | 0.710 | 0.703 |
| CMI | 0.766 (0.701–0.830) | 0.089 | 0.38 | 0.710 | 0.730 |
| 40–49 years (n=143) | | | | | |
| METS-IR | 0.825 (0.754–0.895) | - | 37.04 | 0.742 | 0.842 |
| TyG-BMI | 0.835 (0.768–0.902) | 0.338 | 215.90 | 0.833 | 0.737 |
| TyG-WC | 0.831 (0.762–0.900) | 0.750 | 743.92 | 0.803 | 0.776 |
| LAP | 0.821 (0.750–0.891) | 0.857 | 29.70 | 0.788 | 0.789 |
| TyG | 0.795 (0.718–0.871) | 0.334 | 8.79 | 0.761 | 0.789 |
| TG/HDL-C | 0.794 (0.717–0.871) | 0.266 | 1.85 | 0.851 | 0.711 |
| VAI | 0.797 (0.721–0.872) | 0.369 | 1.39 | 0.788 | 0.776 |
| CMI | 0.808 (0.734–0.882) | 0.491 | 0.44 | 0.833 | 0.737 |
| 50–60 years (n=187) | | | | | |
| METS-IR | 0.781 (0.710–0.853) | - | 35.52 | 0.788 | 0.675 |
| TyG-BMI | 0.773 (0.700–0.846) | 0.537 | 211.57 | 0.848 | 0.608 |
| TyG-WC | 0.758 (0.684–0.833) | 0.356 | 789.48 | 0.667 | 0.792 |
| LAP | 0.746 (0.671–0.820) | 0.170 | 37.12 | 0.621 | 0.808 |
| TyG | 0.704 (0.625–0.784) | 0.034 | 8.81 | 0.591 | 0.760 |
| TG/HDL-C | 0.690 (0.609–0.771) | 0.006 | 2.77 | 0.470 | 0.860 |
| VAI | 0.678 (0.596–0.760) | 0.004 | 1.60 | 0.561 | 0.800 |
| CMI | 0.709 (0.629–0.788) | 0.013 | 0.57 | 0.545 | 0.808 |

IR was defined as HOMA-IR ≥ 3.17, corresponding to the 75th percentile in the overall reference group of normal-weight participants with normal glucose tolerance. *P* values were calculated using DeLong’s nonparametric test to compare AUCs of other evaluated indices with METS-IR. Cut-offs were determined separately within each age subgroup by maximizing the Youden index. METS-IR, metabolic score for insulin resistance; TyG, triglyceride–glucose; TyG-BMI, TyG-body mass index; TyG-WC, TyG-waist circumference; LAP, lipid accumulation product; TG/HDL-C, triglyceride-to-HDL-C ratio; VAI, visceral adiposity index; CMI, cardiometabolic index.

## 1.4 Supplementary Table 4. Comparison of eight IR surrogate indices for identifying Matsuda index-defined IR across age groups.

| Index | AUC (95% CI) | *P* value vs METS-IR | Cut-off | Sensitivity | Specificity |
| --- | --- | --- | --- | --- | --- |
| 20–29 years (n=167) | | | | | |
| METS-IR | 0.781 (0.712–0.851) | - | 32.00 | 0.685 | 0.769 |
| TyG-BMI | 0.807 (0.741–0.873) | 0.025 | 193.21 | 0.663 | 0.846 |
| TyG-WC | 0.784 (0.716–0.853) | 0.886 | 696.33 | 0.517 | 0.910 |
| LAP | 0.804 (0.739–0.869) | 0.350 | 13.23 | 0.764 | 0.718 |
| TyG | 0.756 (0.683–0.829) | 0.494 | 8.36 | 0.652 | 0.795 |
| TG/HDL-C | 0.725 (0.648–0.802) | 0.086 | 1.48 | 0.640 | 0.782 |
| VAI | 0.743 (0.669–0.817) | 0.280 | 0.96 | 0.652 | 0.795 |
| CMI | 0.750 (0.676–0.824) | 0.276 | 0.23 | 0.753 | 0.692 |
| 30–39 years (n=213) | | | | | |
| METS-IR | 0.797 (0.737–0.857) | - | 35.83 | 0.625 | 0.861 |
| TyG-BMI | 0.799 (0.739–0.859) | 0.852 | 204.88 | 0.721 | 0.787 |
| TyG-WC | 0.810 (0.752–0.869) | 0.398 | 688.09 | 0.767 | 0.778 |
| LAP | 0.814 (0.756–0.871) | 0.373 | 22.14 | 0.728 | 0.806 |
| TyG | 0.777 (0.713–0.840) | 0.467 | 8.64 | 0.644 | 0.853 |
| TG/HDL-C | 0.783 (0.720–0.846) | 0.556 | 1.88 | 0.683 | 0.807 |
| VAI | 0.780 (0.717–0.842) | 0.517 | 1.74 | 0.495 | 0.963 |
| CMI | 0.801 (0.740–0.861) | 0.860 | 0.44 | 0.650 | 0.861 |
| 40–49 years (n=143) | | | | | |
| METS-IR | 0.826 (0.755–0.897) | - | 34.86 | 0.853 | 0.743 |
| TyG-BMI | 0.838 (0.771–0.904) | 0.220 | 217.59 | 0.809 | 0.784 |
| TyG-WC | 0.829 (0.762–0.897) | 0.855 | 726.49 | 0.824 | 0.716 |
| LAP | 0.816 (0.745–0.886) | 0.667 | 27.60 | 0.824 | 0.743 |
| TyG | 0.773 (0.693–0.852) | 0.110 | 8.79 | 0.725 | 0.770 |
| TG/HDL-C | 0.778 (0.699–0.857) | 0.107 | 1.85 | 0.812 | 0.703 |
| VAI | 0.782 (0.704–0.859) | 0.160 | 1.39 | 0.750 | 0.757 |
| CMI | 0.792 (0.716–0.868) | 0.191 | 0.44 | 0.794 | 0.716 |
| 50–60 years (n=187) | | | | | |
| METS-IR | 0.747 (0.673–0.822) | - | 35.31 | 0.773 | 0.649 |
| TyG-BMI | 0.723 (0.645–0.800) | 0.044 | 223.80 | 0.653 | 0.730 |
| TyG-WC | 0.705 (0.627–0.783) | 0.067 | 788.53 | 0.613 | 0.784 |
| LAP | 0.691 (0.612–0.770) | 0.033 | 37.50 | 0.560 | 0.811 |
| TyG | 0.666 (0.584–0.749) | 0.028 | 8.92 | 0.467 | 0.848 |
| TG/HDL-C | 0.677 (0.597–0.758) | 0.041 | 2.96 | 0.413 | 0.920 |
| VAI | 0.660 (0.578–0.743) | 0.018 | 1.60 | 0.533 | 0.811 |
| CMI | 0.684 (0.604–0.765) | 0.040 | 0.69 | 0.440 | 0.910 |

IR was defined as a Matsuda index ≤ 3.33, corresponding to the 25th percentile in the overall reference group of normal-weight participants with normal glucose tolerance. *P* values were calculated using DeLong’s nonparametric test to compare AUCs of other evaluated indices with METS-IR. Cut-offs were determined separately within each age subgroup by maximizing the Youden index. METS-IR, metabolic score for insulin resistance; TyG, triglyceride–glucose; TyG-BMI, TyG-body mass index; TyG-WC, TyG-waist circumference; LAP, lipid accumulation product; TG/HDL-C, triglyceride-to-HDL-C ratio; VAI, visceral adiposity index; CMI, cardiometabolic index.

# 2 Supplementary Figures

## 2.1 Supplementary Figure 1. Spearman rank correlations of the Matsuda index calculated using 3-point, 4-point, and 5-point OGTT data and HOMA-IR.

Matsuda-3P, Matsuda-4P, and Matsuda-5P represent calculations using three points (0, 60, and 120 min), four points (0, 30, 60, and 120 min), or five points (0, 30, 60, 120, and 180 min), respectively.

## 2.2 Supplementary Figure 2. Dose–response relationships between IR surrogate indices and IR defined using the Matsuda index.

Restricted cubic splines were used to evaluate dose–response relationships between IR indicated by the Matsuda index and the following: METS-IR (A), TyG (B), TyG-BMI (C), TyG-WC (D), LAP (E), TG/HDL-C (F), VAI (G), and CMI (H). The model was adjusted for age, sex, current smoker and drinker status, SBP, DBP, and antihypertensive and lipid-lowering medication use.

## 2.3 Supplementary Figure 3. Comparison of the discriminative performance of eight surrogate indices for IR using a published HOMA-IR threshold of 2.69.

ROC curves evaluated the discriminative performance of eight insulin-independent surrogate indices, with IR defined as HOMA-IR ≥ 2.69, in the overall population.
